# Repeated Muscle Injury as a Presumptive Trigger for Chronic Masticatory Muscle Pain

**DOI:** 10.1155/2011/647967

**Published:** 2011-06-12

**Authors:** Dean Dessem, Richard M. Lovering

**Affiliations:** ^1^Department of Neural and Pain Sciences, University of Maryland, 650 West Baltimore Street, Baltimore, MD 21201, USA; ^2^Graduate Program in Life Sciences, University of Maryland, 650 West Baltimore Street, Baltimore, MD 21201, USA; ^3^Department of Orthopaedics, University of Maryland, 20 Penn Street, Baltimore, MD 21201, USA

## Abstract

skeletal muscles sustain a significant loss of maximal contractile force after injury, but terminally damaged fibers can eventually be replaced by the growth of new muscle (regeneration), with full restoration of contractile force over time. After a second injury, limb muscles exhibit a smaller reduction in maximal force and reduced inflammation compared with that after the initial injury (i.e., repeated bout effect). In contrast, masticatory muscles exhibit diminished regeneration and persistent fibrosis, after a single injury; following a second injury, plasma extravasation is greater than after a single injury and maximal force is decreased more than after the initial injury. Thus, masticatory muscles do not exhibit a repeated bout effect and are instead increasingly damaged by repeated injury. We propose that the impaired ability of masticatory muscles to regenerate contributes to chronic muscle pain by leading to an accumulation of tissue damage, fibrosis, and a persistent elevation and prolonged membrane translocation of nociceptive channels such as P2X_3_ as well as enhanced expression of neuropeptides including CGRP within primary afferent neurons. These transformations prime primary afferent neurons for enhanced responsiveness upon subsequent injury thus triggering and/or exacerbating chronic muscle pain.

## 1. Introduction

Musculoskeletal pain is estimated to afflict 15% of the population, is one of the most frequent symptoms encountered by primary care providers [1, 2], and comprises a substantial portion of the total cost of illness [1–3]. Muscle pain is a prominent component in many musculoskeletal disorders, including low-back pain, tension-type headache, fibromyalgia and whiplash [4–6]. In the craniofacial region, temporomandibular disorders (TMD) affect 4–12% of the population (~35 million in the United States), with masticatory muscle pain being the most frequent (66%) patient complaint [7]. TMDs are often not restricted to the temporomandibular joint, but frequently include pain and tenderness of the masticatory muscles [4–6] designated as Group I in the Research Diagnostic Criteria for TMD [8]. It is estimated that one-half of TMD cases are these masticatory myalgias [9]. Patients with TMD frequently also have fibromyalgia [10–12], a musculoskeletal disorder characterized by widespread musculoskeletal pain and diffuse muscle tenderness [11]. Approximately 2–5% of the population meet the diagnostic criteria for fibromyalgia [13, 14]. The similarities of TMD and fibromyalgia have lead to speculation that these conditions may involve common mechanisms of muscle pain with different durations [15, 16]. While little is known about the mechanisms underlying muscle pain, available data indicate that the mechanisms underlying muscle pain differ from those underlying cutaneous or visceral pain (for review see [17, 18]). 

In spite of the prevalence of muscle pain, current therapies for muscle pain are often ineffective and can even be dangerous [19]. For instance, NSAIDS and COX-2 drugs are no more effective than placebo in treating some types of muscle pain and have substantial risks [20–25]. Weak opioids (e.g., codeine, tramadol) do not alleviate pain produced by muscle injury [23, 26]. More powerful opioids such as hydrocodone, morphine, and oxycodone can reduce chronic pain, but have many deleterious effects [27–29]. Thus it is important to understand the mechanisms of muscle pain in order to develop new, effective therapeutic strategies for muscle pain.

Pain resulting from muscle disorders can be persistent, although the mechanisms by which this chronic pain becomes established are not understood. Patients with TMD and fibromyalgia exhibit altered central nociceptive processing [30–33], which is hypothesized to be generated by a peripheral trigger [34]. Nociceptive input from muscle afferents is particularly potent at generating CNS wind-up [35]. Recent muscle pain studies support the involvement of peripheral stimuli in chronic muscle pain by demonstrating that enhanced central pain processing in fibromyalgia is maintained by muscle afferent input [36, 37]. We predict that comparable processes exist in muscle-based TMDs, given the similar characteristics of fibromyalgia and muscle-based TMDs [15, 16]. In this communication, we explore the potential for muscle injury to contribute to the triggering and/or maintenance of chronic pain.

## 2. Typical Experimental Methods of Producing Muscle Pain Do Not Accurately Model Muscle Pain

In spite of its prevalence, currently there are no widely accepted models of muscle pain, and most methods used to investigate muscle pain do not accurately reproduce the features of pain reported in humans suffering from muscle pain. For instance, injection of exogenous substances such as complete Freund's adjuvant (CFA) [38–40] has been used to evoke inflammatory muscle pain. However, CFA produces a massive inflammatory response with large intramuscular vacuoles and enormous inflammatory cell infiltration [38], characteristics that differ so dramatically from those reported in muscle pain patients [41] that adjuvant injection has only very limited relevance for studies of muscle pain. While injection of hypertonic saline produces sensations that mimic muscle pain [42, 43], hypertonic saline activates both muscle nociceptors [44, 45] and nonnociceptors [46, 47] and does not alter muscle lactate or PGE_2_ as reported in muscle pain [45]. Acidic saline injected into limb muscles activates some muscle afferents [47] and produces hyperalgesia [48–50]. Although ASIC3 (acid-sensing ion channels) are present on craniofacial muscle afferents [51, 52], injection of acidic saline into the masseter does not produce hyperalgesia or alter calcitonin gene-related peptide (CGRP) and substance P expression [51]. Acid saline, therefore, is a valuable model for limb, but not craniofacial muscle pain. Injection of the polysaccharide carrageenan activates fine muscle afferents and produces hyperalgesia [53–55], but does not elevate intramuscular TNF-*α* [23]. Since TNF-*α* is elevated in TMD and fibromyalgia [56–58], carrageenan is not an accurate model for TMD and fibromyalgia. Mustard oil and capsaicin both inflame tissue including muscle [59–64]. Mustard oil acts via TRP (transient receptor potential channel) A1 channels on a subset of unmyelinated fibers [65] while capsaicin acts via TRPV1 channels on a subset of unmyelinated muscle afferents [66]. Since algogens do not activate thinly myelinated muscle afferents, they do not activate the spectrum of afferent types that participate in muscle pain [67–69]. Endogenous substances have also been injected into muscles. Serotonin, bradykinin, ATP, TNF-*α*, and NGF activate a subpopulation of muscle afferents and produce pain [47, 52, 70–77]. Single substances (5-HT, bradykinin, etc.) interact with subsets of nociceptors providing insight into specific aspects of nociception; however, they cannot simulate muscle pain that involves multiple nociceptive channels and afferent types. Even if multiple algogens are used [73, 78], the environment at nerve terminals during pain cannot be reproduced due to differences in spatial application of the algogen, diffusion, and the fact that different mediators do not appear simultaneously in inflammation. For these reasons, we concentrate in this communication on data derived from eccentric (lengthening) muscle contractions, a noninvasive, physiologically relevant *in vivo* model of producing muscle pain and inflammation. This model of muscle strain injury incorporates movement and contraction, fundamental properties of muscle [79, 80].

When an activated muscle is forced to lengthen because the external load exceeds the tension generated by the muscle contraction, this is termed a lengthening `eccentric' contraction. Although eccentric contractions require less energy, the force generated during a maximal eccentric contraction is about double the force developed during a maximal isometric contraction; therefore eccentric contractions are more likely to produce damage than either isometric or concentric (shortening) contractions. Using an animal model, forceful eccentric muscle contraction uniquely disrupts selected myofibers [79, 81], comparable to the selective myofiber damage in humans after eccentric exercise [82]. As few as 12 unaccustomed voluntary eccentric muscle contractions can produce muscle pain often referred to as delayed onset muscle soreness (DOMS) [83]. Furthermore, inflammation evoked by muscle contraction typically develops more slowly than the pulsatile inflammation produced by injection of algogens such as CFA, which evoke changes in neuropeptide mRNA within 30 min [38]. Eccentric muscle contractions can be produced in the laboratory setting by manually lengthening a muscle during electrical stimulation [84, 85]. Only a few contractions are needed to induce readily detectable muscle inflammation [84, 86]. Both voluntary and stimulus-induced eccentric muscle contraction as well as rapid muscle stretching damage a subpopulation of myofibers [81, 82, 87, 88], produce muscle pain and soreness [45, 85], evoke myonecrosis, induce inflammatory infiltration, elevate inflammatory proteins [81, 84, 89, 90], decrease muscle force and range of motion (for review [91]), and activate genes associated with muscle repair and apoptosis [92]. Intramuscular calcitonin gene related peptide (CGRP) and vascular endothelial growth factor (VEGF), a proangiogenic cytokine which increases after exercise [93–95], also increase after eccentric muscle contraction [81]. While in this paper we present data derived from eccentric contraction produced by muscle lengthening following supramaximal muscle contraction, comparable, but smaller effects are observed following submaximal eccentric contractions and behaviors such as downhill running [90, 96].

## 3. Injury Evokes Differential Effects in Muscles from Various Body Regions

Repair and regeneration in hindlimb muscle following injury involves activation of satellite cells within 24–48 hours [97]. These mononuclear cells are situated outside the sarcolemma, but inside the basement membrane of each muscle fiber. They are normally quiescent, however they are thought to become active with stimulation (e.g., injury). Under appropriate conditions, satellite cells develop into myoblasts, which fuse to form myotubes [98]. Myotubes can then repair, or even replace, damaged muscle fibers. It is generally hypothesized that satellite cells, after several rounds of proliferation, are a determinant factor in the functional recovery of muscle. Within 7–14 days following injury, myofibers are approaching normal size [99] and myofibers return to normal by 24 days [100]. Evidence indicates that the response to eccentric contraction differs between hindlimb and forelimb muscles. Blood creatine kinase levels and muscle soreness are reported to be greater following muscle damage to forelimb compared to hindlimb muscles [101]. Recovery of function after injury is also reported to be slower in forelimb versus hindlimb muscles [101]. In fact, when direct comparisons are made using similar indices of muscle damage, creatine kinase levels are greater after forelimb eccentric contraction and muscle recovery is longer for forelimb muscles [102].

Masticatory muscle responds very differently to injury than hindlimb muscle. Twelve days following muscle injury produced by a single crush or freezing injury, large areas of muscle exhibit minimal evidence of muscle regeneration [98]. Following a similar injury, hindlimb muscle shows centrally nucleated fibers (CNFs), indicative of regenerating muscle. At 19–21 days following injury, masseter muscle regeneration is still impaired and the masseter muscle exhibits extensive interstitial connective tissue. Even 45 days following a single injury, regeneration of the masseter muscle is less extensive than observed in hindlimb muscle 12 days after injury [98].

We have observed comparable findings after muscle injury produced by a single bout of eccentric muscle contractions [81]. Adult, male Sprague Dawley rats were used for all experiments. Animals received humane care in compliance with the *Guide for the Care and Use of Laboratory Animals* (NIH publication no. 86–23, revised 1985) and the Use Committee and the Committee for Research and Ethical Issues of the IASP. All laboratory procedures were reviewed and approved by the University of Maryland Animal Care and Use Committee and every effort was made to minimize any suffering. We first anesthetized the skin overlying the masseter muscle by applying a topical anesthetic (2.5% lidocaine, 2.5% prilocaine). We used a combination of lidocaine and procaine because this eutectic mixture has been shown to produce more effective cutaneous anesthesia in humans than either substance alone [103]. After two hours, when topical anesthesia was well established, rats were anesthetized with isoflurane. We have previously shown that lidocaine/prilocaine cream produces cutaneous anesthesia in the rat facial skin at this time [104]. A rod coupled to a stepping motor and potentiometer was then positioned in the diastema of the mandible. To produce eccentric contraction of the masseter muscle, we used an established *in vivo* model previously described for the hindlimb [79, 80, 105]. The masseter was contracted by electrical stimulation (1 s trains, 100 Hz, 0.3 ms pulse at 0.3 Hz) using surface electrodes. Stimulation current was adjusted (5–12 mA) to produce a supramaximal muscle contraction. Neurogenic plasma extravasation was prevented by anesthetising the skin overlying the muscle [81] and using a high-frequency stimulation regime, which does not activate group III and IV masseter muscle afferent axons [106]. Eccentric muscle contraction was produced by displacing the mandible 25 degrees of jaw opening at a rate of 0.6°/ms 150 milliseconds into a maximal muscle contraction. Mandibular displacement was produced using a stepping motor (1.8°/step NMB Technologies, Chatsworth, CA) controlled by a custom LabVIEW program (LabVIEW, version 8.5 National Instruments, Austin, TX). Muscle torque was measured using a torque sensor (model QWLC-8 M Sensotec, Columbus OH) and amplifier (model DV-05, Sensotec). Angular displacement of the mandible was monitored with a potentiometer. Displacement, angular position, and torque were synchronized using a custom LabVIEW program. Signals were sampled at 2 KHz using a 16-bit analog-to-digital converter (PCI-6221, National Instruments). The eccentric muscle contraction regime consisted of 5 sets of 15 eccentric muscle contractions (75 total contractions) with a five-minute rest between sets.

**Figure 1:**
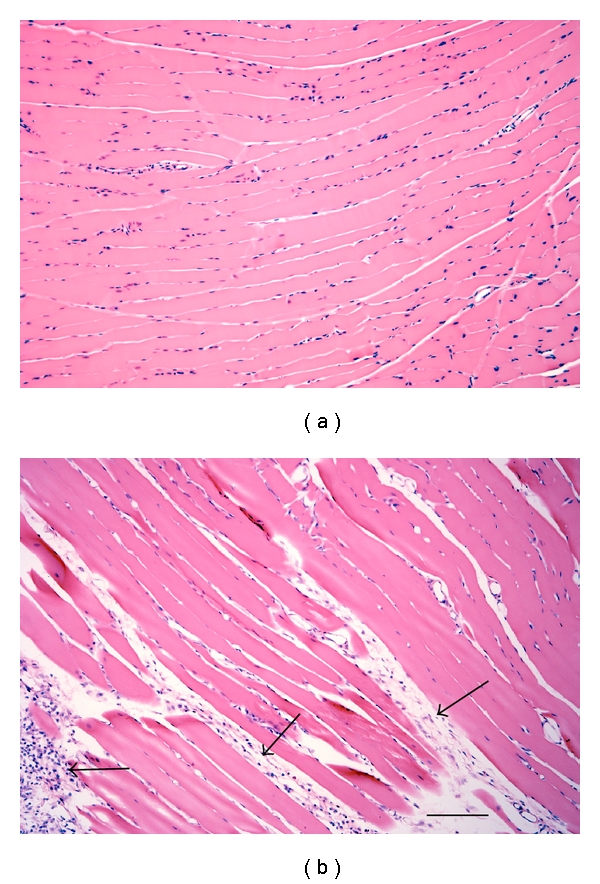
Photomicrograph of the masseter muscle stained with H&E. (a) Naive masseter muscle. (b) Masseter muscle 12 days after eccentric muscle contraction. Arrows indicate regions of fibrosis. Scale bar = 100 *μ*m.

Muscle regeneration was not evident 32 days after one bout of eccentric contraction of the masseter muscle [107] and considerable fibrosis was present (Figure 1). These characteristics correspond to the impaired regeneration and extensive fibrosis evident for at least 45 days after crush or freeze injury to the masseter muscle [98]. In contrast to the masseter muscle, hindlimb muscles such as the tibialis anterior regenerate in 7–12 days after crush or freeze injury [98] and 5–14 days after eccentric muscle contraction [105, 108]. 

**Figure 2:**
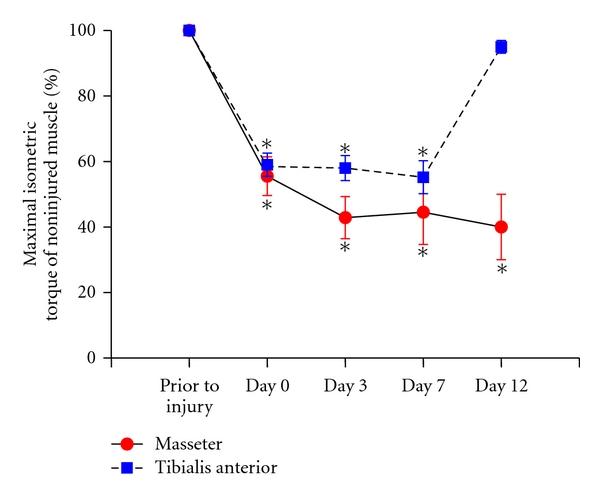
Maximal isometric torque produced before and at various times after eccentric muscle contraction. Solid line is masseter muscle and dashed line is tibialis anterior muscle. Note equivalent loss of force for Masseter and tibialis anterior at day 0 after eccentric contraction. Asterisks denote significant reductions from initial maximal torque. masseter = 60 eccentric contractions, tibialis anterior = 150 eccentric contractions, masseter *n*  =  6, and tibialis anterior *n*  =  25.

We operationally defined muscle injury as a loss in the ability of the muscle to produce force. Torque of a muscle is represented by the equation *T* = *F*∗*d*, where *T *is torque, *F* is muscle force, and *d *is the moment arm of the muscle. Because we use a maximal tetanic contraction and we measured torque at a fixed position, our measure of torque ultimately reflects muscle force. Maximal contractile force is a strong indicator of the overall status of a muscle [109] and a reliable indicator of injury [110, 111]. Therefore, we investigated loss of maximal torque following injury in masticatory and hindlimb muscles. A variety of contraction schemes were tested, and we found that 60 masseter eccentric contractions (0.6°/ms) produce a 43% reduction in maximal torque measured at resting length (L_0_) 10 minutes after contraction (Figure 2, arrow *n*  =  6 rats). For the tibialis anterior muscle (*n*  =  25), 150 eccentric contractions produced in an analogous manner resulted in a 41% deficit in maximal torque (Figure 2, asterisk [105]). Thus, to produce a comparable loss of isometric force following a single bout of eccentric muscle contraction, less than one-half as many eccentric contractions of the masseter muscle were needed than for the tibialis anterior muscle (Figure 2). These data suggest that the loss of contractile force in the masseter after injury evoked by a single bout of eccentric muscle contractions is greater than in hindlimb muscles. Much less information is available on the effects of injury on muscles from other parts of the body such as the back and neck which may have profound significance for musculoskeletal pain disorders. It will be particularly important for future studies to determine the effects and functional significance of injury on muscles from other regions of the body, such as the back and neck, which may have profound significance for musculoskeletal pain disorders.

## 4. Masticatory Muscles Do Not Exhibit a Repeated Bout Effect

In limb muscles, lengthening contractions are associated with injury, but they can also provide significant protection against future injury. Compared to the first bout, a second bout of lengthening contractions in hindlimb and forelimb muscles is associated with a decreased loss of contractile force, less soreness, and a reduction in the amount of muscle proteins in the blood. However, little is known about the conditions that result in the protective adaptation [108, 112–117]. This adaptive effect is often referred to as the repeated bout effect (RBE) and has been demonstrated in both animals and humans (for review see [118]). While a number of mechanisms have been proposed to underlie the RBE including neuronal, cellular, and mechanical adaptations, the processes involved in the RBE are still not well established. Neuronal mechanisms, such as changes in motor unit recruitment, have been proposed. Although there is some evidence for changes in motor unit recruitment following injury, the RBE can be evoked by electrical stimulation [113], indicating that changes in motor unit recruitment alone are not sufficient to account for the repeated bout effect. 

The RBE has also been attributed to cellular mechanisms, including change in the number of sarcomeres, excitation-contraction coupling, and/or inflammation. An increase in the number of sarcomeres has been reported following eccentric exercise [119–121]. However, the RBE can also be demonstrated following a minimal stimulus, such as a few eccentric contractions, or passive stretching, a stimulus that may be insufficient to evoke sarcomere remodeling [118]. While excitation-contraction coupling can be disrupted immediately following eccentric contraction [122], it does not correspond to the timing of loss of strength in humans several days following a repeated bout of eccentric contraction [112]. Inflammation typically occurs following eccentric muscle contraction [86, 90], and this inflammatory response is reduced following a subsequent bout of eccentric hindlimb or forelimb muscle contraction [114, 123]. It has been proposed that inflammation may help to provide a protective function from damage after subsequent bouts of eccentric muscle contraction [123, 124]. Myofiber damage produced by a bout of eccentric muscle contractions is reduced after subsequent bouts [125]. Thus, it is difficult to determine if the reduced inflammation that occurs following a repeated bout of contractions is a primary process, or reduced due to diminished tissue injury. However, it has been shown that passive stretching and concentric muscle contraction, processes that do not produce overt tissue damage evident at the light microscopical level, can evoke a small repeated bout effect [126]. Alteration in the mechanical properties of muscle including muscle stiffness and altered expression of cytoskeletal proteins have also been postulated to contribute to the RBE. While passive muscle stiffness increases following eccentric exercise [127], it is unclear that this increases the susceptibility of the muscle to injury [128]. Thus while several of these mechanisms may contribute to the RBE, the precise mechanisms of the repeated bout effect remain elusive.

**Figure 3:**
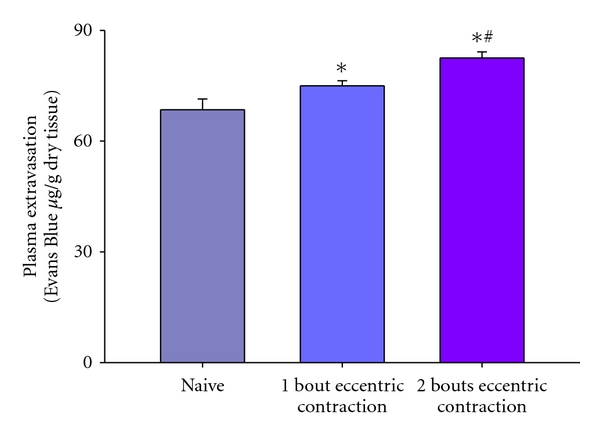
Plasma extravasation after one and two bouts of eccentric contraction of the masseter muscle. Note the increased plasma extravasation after two bouts of eccentric contraction. Asterisk denotes significant difference from naïve and number sign denotes significant difference from 1 bout.

**Figure 4:**
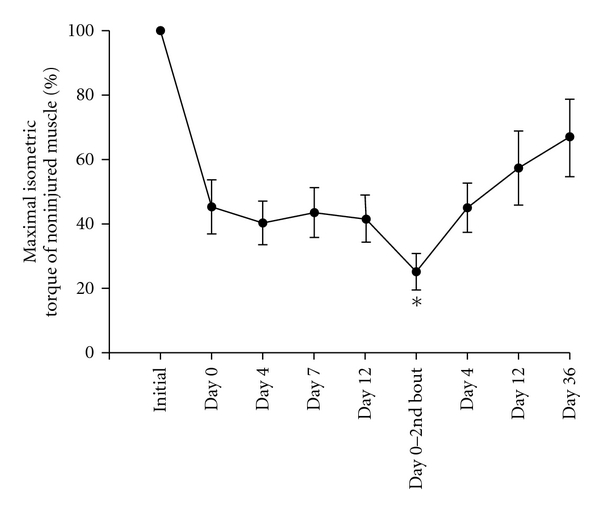
Effect of repeated masseter muscle injury on maximal isometric torque. Asterisk denotes significant difference from maximal torque after a single bout of eccentric contraction (day 0 initial bout).

**Figure 5:**
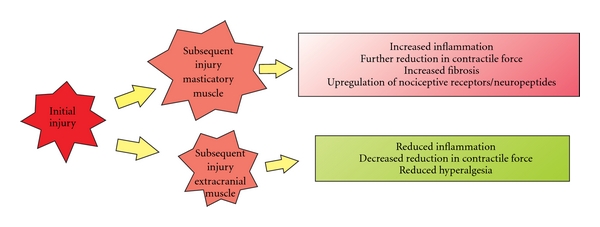
Diagrammatic representation of the response of masticatory versus limb muscle to repeated injury.

Little is known about the effects of repeated injury on craniofacial muscle, therefore we have begun to examine repeated injury of the masseter muscle. Craniofacial muscle has distinct origins and developmental regulatory mechanisms from that of limb muscle. The masseter is derived from the first pharyngeal (branchial) arch and has been shown to respond differently to muscle injury [98]. The effect of impaired regeneration and fibrosis of the masseter muscle after repeated injury was initially investigated by examining plasma extravasation defined here as (wet muscle weight − dry muscle weight/wet muscle weight) × 100, as an index of muscle edema [81]. Muscle edema significantly increased not only after one bout of eccentric contraction compared to naive (Figure 3 asterisk) but also after two bouts of contraction spaced 12 days apart (Figure 3 number sign). Note that muscle edema increased significantly after two bout compared to one bout of contraction indicating a lack of repeated bout effect (naive *n*  =  4, 1 bout *n*  =  4, 2 bouts *n*  =  6, *P* < .025 for 2 bouts versus 1 bout and  .049 for 1 bout versus naive, ANOVA followed by Holm-Sidak method, Figure 3). Mechanical hyperalgesia was also measured by determining the threshold for a head withdrawal reflex [81]. Animals were initially habituated to stand unrestrained on their hindpaws and lean on the tester's hand covered with a leather glove. Mechanical thresholds were then determined by probing the masseter muscle through the facial skin using a rigid von Frey filament coupled with a force transducer with a fixed contact area (Electrovonfrey, model no 2290, IITC Inc). The force needed to produce a withdrawal of the head was recorded following five stimulus presentations at one minute intervals. The mean values of the five readings was used for analysis. Using this method, mechanical hyperalgesia was found to be more profound and persisted for at least 7–14 days longer after multiple bouts of eccentric contraction of the masseter muscle than one bout (ANOVA, *P* < .05, *n*  =  7). Taken together, these data contrast strongly with data derived from hindlimb muscles, which show a RBE in regards to inflammation and muscle soreness [112, 113, 129]. We also examined the effects of two bouts of eccentric muscle contraction spaced 12 days apart on masseter contractile function by measuring torque in 7 male rats. After a second bout of eccentric contraction, masseter maximal torque decreased by 79% compared to the initial maximal torque at day 0, and decreased by an additional 60% compared to maximal torque immediately prior to the second bout of eccentric contractions (Figure 4). These data show that a second bout of eccentric contractions of the masseter muscle further reduces muscle force (Mann-Whitney rank sum test, *n*  =  7 animals per group, *P* = .026) in contrast to the tibialis anterior muscle in which a second bout of eccentric muscle contraction results in very little or no further reduction in muscle force (i.e., repeated bout effect) [108, 113]. Thus, the masseter muscle not only lacks a repeated bout effect, but instead sustains increased damage upon repeated bouts of muscle injury (Figure 5). We propose this difference evokes mechanisms that contribute to chronic craniofacial muscle pain.

## 5. Synthesis of the Role of Muscle Injury in Chronic Muscle Pain Including Potential Therapeutic Targets

In this communication we show that repeated bouts of injury to masticatory muscles do not evoke the adaptive RBE present in limb muscles, but rather compound muscle injury. Patients with TMD and fibromyalgia exhibit altered central nociceptive processing [30–33, 37], which is most likely initially triggered from a peripheral source [34]. Nociceptive input from muscle afferent neurons is particularly potent at generating central nervous system wind-up [35]. One potential source for muscle injury is oral parafunctional behaviors. Evidence shows that oral parafunctional behaviors that increase muscle tension are good predictors of orofacial pain levels in patients with TMD [130, 131]. It is also known that experimental bruxism produces muscle soreness, described as moderate muscle pain that is exacerbated by movement [132, 133]. Horizontal jaw movement at 50% maximum voluntary contraction for 5 min, or jaw protrusion and retrusion under load, also produces delayed jaw muscle pain [134]. Finally, eccentric, but not concentric, contraction of the masseter muscle lowers the masseter pressure pain threshold [135, 136], which is consistent with the greater myofiber damage produced by eccentric contractions [137]. Since parafunctional behaviors can occur for prolonged periods, we propose that the impaired ability of masticatory muscles to regenerate results in neuronal transformations. This in turn chronically enhances the responsiveness of muscle primary afferent neurons to subsequent injury and thus serves as a source to initiate and/or exacerbate chronic muscle pain.

Nerve growth factor (NGF) is a homodimeric protein that binds to TrkA and p75 receptors [138, 139] and is implicated in mechanical and heat hyperalgesia [140–143]. Several findings establish a role for NGF in peripheral muscle pain mechanisms. First, NGF levels in the masseter muscle of patients with TMD are negatively correlated with pressure pain threshold and positively correlated with descriptors of pain [144]. Levels of NGF are also elevated in inflamed muscle following injury including injury evoked by eccentric muscle contraction [145–147]. While other studies implicate NGF in muscle pain [77, 148, 149], high concentrations of NGF were used. Several sources for intramuscular NGF exist. Adult myofibers do not produce NGF [150], although developing and dystrophic myofibers can [150, 151]. Other potential sources for NGF include keratinocytes [152], fibroblasts [153], and mast cells [154–156]. NGF is known to upregulate many nociceptive channels and neuropeptides implicated in muscle pain [157–161], and, therefore, we propose that NGF is not only involved in acute muscle pain, but plays an instrumental role in chronic muscle pain by sustained modulation of nociceptive channel and neuropeptide expression. Among these, we expect that P2X_3_ and CGRP are particularly important for deep tissue craniofacial pain. 

P2X receptors comprise a family of ionotropic receptors which are activated by ATP (for review [162, 163]). In muscle, injection of ATP elicits pain [73] and activates muscle nociceptors [164]. Nonspecific P2X antagonists also reduce nocifensive behavior following muscle pain [165]. One member of the P2X family, the P2X_3_ receptor, is specifically implicated in nociception [166]. Since a much higher percentage of craniofacial muscle afferent neurons express P2X_3_ than limb muscle afferent neurons [167], P2X_3_ is particularly implicated in craniofacial deep tissue pain. P2X_3_ receptors are present on masseter muscle afferent neurons [168] and rapidly desensitizing currents characteristic of P2X_3_ receptors can be activated in a subpopulation of masseter muscle afferents by applying ATP [52]. P2X_3_ immunopositive muscle afferent neurons are increased 15 days following repetitive muscle contraction and rapid stretching [81]. Thus, physiologically relevant stimuli upregulate P2X_3_ in primary muscle afferent neurons for prolonged periods of time. One potential source of ATP to activate P2X_3_ receptors is ATP released from the cytosol of damaged cells. In coculture systems, action potentials and inward currents are evoked in nociceptors when nearby cells are mechanically damaged, and these responses are demonstrated to be mediated by ATP [169]. In muscle, the concentration of ATP within myofibers is approximately 10 mM [170], a concentration that readily activates muscle primary afferent neurons *in vivo *[164], demonstrating that sufficient ATP is present within myofibers to activate muscle afferent neurons. Since eccentric muscle contraction mechanically damages myofibers and disrupts their membrane [81], we propose that ATP is released from damaged myofibers following muscle injury and activates P2X_3_ receptors on muscle nociceptors.

Considerable evidence implicates the neuropeptide, calcitonin gene-related peptide (CGRP) in nociception and inflammation [171–175]. CGRP is a 37 amino acid neuropeptide synthesized in primary afferent neurons. CGRP is a potent vasodilator of blood vessels [171, 176] including those in muscles [176], and mediates neurogenic inflammation [177]. CGRP has been implicated specifically in nociceptive mechanisms from deep tissues [178], including muscle [54] and intramuscular CGRP is significantly increased following muscle injury evoked by eccentric muscle contractions [94]. Seventy-five percent of masseter P2X_3_ muscle afferents colocalize CGRP [168]. We predict that this extensive colocalization indicates greater interaction between CGRP and P2X_3_ in trigeminal, compared to dorsal root ganglion neurons, where neuropeptides and P2X_3_ are segregated [179, 180]. NGF not only upregulates CGRP [181], but also P2X_3_ [157, 159, 182]. We propose that increased intramuscular NGF following myofiber injury and muscle inflammation not only upregulates CGRP, but also increases P2X_3_ expression in muscle primary afferent neurons priming the responsiveness of these neurons upon subsequent injury.

Additional factors to consider are that stress and autonomic dysfunction are correlated with some muscle pain disorders [183–187]. When stress is combined with eccentric contractions of hindlimb muscles, allodynia persists for up to 35 days and becomes bilateral [188]. This finding demonstrates that muscle injury can evoke long-lasting neuronal plasticity. Thus, we predict that acute muscle injury, particularly when combined with stress, can evoke central nervous system changes after which pain can become independent of peripheral drive, and that intermittent muscle injury exacerbates pain even after central pain transformations have occurred. Little is known about potential interactions between muscle injury, autonomic dysfunction, and the development of chronic muscle pain, making it an important area for future research.

We hypothesize that peripheral mechanisms involving primary afferent neurons from deep tissues are instrumental in the development of central nervous system transformations, such as central sensitization that occurs in muscle-based TMDs and fibromyalgia [30–32, 189]. Thus, agents capable of reducing primary afferent drive evoked by muscle inflammation have potential as acute therapeutics and as modulators of long-term nociceptive phenomena. We propose that increased intramuscular NGF after muscle injury plays a critical role in chronic pain by persistently upregulating P2X_3_ and CGRP in muscle primary afferent neurons. Although selective P2X_3_ antagonists exist [190], rather than directly targeting P2X_3_, a potentially more powerful approach is to concentrate on CGRP antagonists and NGF biologics (for review [191, 192]), because these agents have the potential to decrease both neurogenic inflammation and P2X_3_ upregulation. We predict that CGRP antagonists will not only reduce vasodilatation and CGRP synthesis and release, but that they will also attenuate the upregulation of P2X_3_ receptors, reducing the activation of muscle nociceptors by ATP. We also anticipate that anti-NGF antibodies will have multiple antinociceptive actions. These include, but are not limited to, blocking NGF-mediated upregulation of CGRP and reducing the upregulation of P2X_3_ due to CGRP.

## 6. Conclusions

In this communication we have described differences in the response to injury of masticatory muscle versus hindlimb muscle. We also included new evidence that masticatory muscles do not adapt to repeated injury as occurs in hindlimb muscle (i.e., masticatory muscles do not exhibit the repeated bout effect). We propose that acute bouts of injury, as occurs during oral parafunctions, increase intramuscular nerve growth factor evoking a persistent upregulation of nociceptive receptors and neuropeptides. This mechanism primes primary afferent neurons for enhanced responsiveness upon subsequent injury and serves to trigger and/or exacerbate chronic muscle pain.

## References

[1] Magni G, Caldieron C, Rigatti-Luchini S, Merskey H (1990). Chronic muscoskeletal pain and depressive symptoms in the general population. An analysis of the 1st National Health and Nutrition Examination Survey data. *Pain*.

[2] Kantor TG (1991). The pharmacological control of musculoskeletal pain. *Canadian Journal of Physiology and Pharmacology*.

[3] Jacobson L, Mariano AJ, Chabal C, Chancy EF, Mar C (1996). What is adequate and appropriate pain treatment?. *Journal of the American Medical Association*.

[4] McNeill C, Mohl ND, Rugh JD, Tanaka TT (1990). Temporomandibular disorders: diagnosis, management, education, and research. *The Journal of the American Dental Association*.

[5] Clark GT, Laskin DM, Greene CS, Hylander WL (2006). Treatment of myogenous pain and dysfunction. *TMDs: An Evidence-Based Approach to Diagnosis and Treatment*.

[6] Epker J, Gatchel RJ, Ellis E (1999). A model for predicting chronic TMD: practical application in clinical settings. *Journal of the American Dental Association*.

[7] Machado LPS, Nery CG, Leles CR, Nery MBM, Okeson JP (2009). The prevalence of clinical diagnostic groups in patients with temporomandibular disorders. *Cranio*.

[8] Dworkin SF, LeResche L (1992). Research diagnostic criteria for temporomandibular disorders: review, criteria, examinations and specifications, critique. *Journal of Craniomandibular Disorders*.

[9] Stohler CS (1999). Muscle-related temporomandibular disorders. *Journal of Orofacial Pain*.

[10] Plesh O, Wolfe F, Lane N (1996). The relationship between fibromyalgia and temporomandibular disorders: prevalence and symptom severity. *Journal of Rheumatology*.

[11] Hedenberg-Magnusson B, Ernberg M, Kopp S (1997). Symptoms and signs of temporomandibular disorders in patients with fibromyalgia and local myalgia of the temporomandibular system: a comparative study. *Acta Odontologica Scandinavica*.

[12] Plesh O, Gansky S, Laskin D, Green C, Hylander W (2006). Fibromyalgia. *TMDs: An Evidence-Based Approach to Diagnosis and Treatment *.

[13] Wolfe F, Ross K, Anderson J, Russell IJ, Hebert L (1995). The prevalence and characteristics of fibromyalgia in the general population. *Arthritis and Rheumatism*.

[14] McLean SA, Clauw DJ (2005). Biomedical models of fibromyalgia. *Disability and Rehabilitation*.

[15] Widmer CG (1997). Idiopathic masticatory muscle pain. *Pain Forum*.

[16] Wolfe F, Katz RS, Michaud K (2005). Jaw pain: its prevalence and meaning in patients with rheumatoid arthritis, osteoarthritis, and fibromyalgia. *Journal of Rheumatology*.

[17] Mense S (1993). Nociception from skeletal muscle in relation to clinical muscle pain. *Pain*.

[18] Mense S, Simons D (2000). *Muscle Pain, Understanding Its Nature, Diagnosis and Treatment*.

[19] Curatolo M, Bogduk N (2001). Pharmacologic pain treatment of musculoskeletal disorders: current perspectives and future prospects. *Clinical Journal of Pain*.

[20] Smith SG (1989). Dangers of NSAIDs in the elderly. *Canadian Family Physician*.

[21] Tramèr MR, Moore RA, Reynolds DJM, McQuay HJ (2000). Quantitative estimation of rare adverse events which follow a biological progression: a new model applied to chronic NSAID use. *Pain*.

[22] Donnelly AE, Maughan RJ, Whiting PH (1990). Effects of ibuprofen on exercise-induced muscle soreness and indices of muscle damage. *British Journal of Sports Medicine*.

[23] Loram LC, Mitchell D, Fuller A (2005). Rofecoxib and tramadol do not attenuate delayed-onset muscle soreness or ischaemic pain in human volunteers. *Canadian Journal of Physiology and Pharmacology*.

[24] Mitchell JA, Lucas R, Vojnovic I, Hasan K, Pepper JR, Warner TD (2006). Stronger inhibition by nonsteroid anti-inflammatory drugs of cyclooxygenase-1 in endothelial cells than platelets offers an explanation for increased risk of thrombotic events. *FASEB Journal*.

[25] Castrillon EE, Cairns BE, Ernberg M (2007). Effect of a peripheral NMDA receptor antagonist on glutamate-evoked masseter muscle pain and mechanical sensitization in women. *Journal of Orofacial Pain*.

[26] Barlas P, Craig JA, Robinson J, Walsh DM, Baxter GD, Allen JM (2000). Managing delayed-onset muscle soreness: lack of effect of selected oral systemic analgesics. *Archives of Physical Medicine and Rehabilitation*.

[27] Jamison RN, Raymond SA, Slawsby EA, Nedeljkovic SS, Katz NP (1998). Opioid therapy for chronic noncancer back pain: a randomized prospective study. *Spine*.

[28] Ray CA, Carter JR (2007). Central modulation of exercise-induced muscle pain in humans. *Journal of Physiology*.

[29] Benyamin R, Trescot AM, Datta S (2008). Opioid complications and side effects. *Pain Physician*.

[30] Maixner W, Fillingim R, Sigurdsson A, Kincaid S, Silva S (1998). Sensitivity of patients with painful temporomandibular disorders to experimentally evoked pain: evidence for altered temporal summation of pain. *Pain*.

[31] Desmeules JA, Cedraschi C, Rapiti E (2003). Neurophysiologic evidence for a central sensitization in patients with fibromyalgia. *Arthritis and Rheumatism*.

[32] Sarlani E, Grace EG, Reynolds MA, Greenspan JD (2004). Evidence for up-regulated central nociceptive processing in patients with masticatory myofascial pain. *Journal of Orofacial Pain*.

[33] Staud R, Robinson ME, Price DD (2005). Isometric exercise has opposite effects on central pain mechanisms in fibromyalgia patients compared to normal controls. *Pain*.

[34] Vierck CJ (2006). Mechanisms underlying development of spatially distributed chronic pain (fibromyalgia). *Pain*.

[35] Wall PD, Woolf CJ (1984). Muscle but not cutaneous C-afferent input produces prolonged increases in the excitability of the flexion reflex in the rat. *Journal of Physiology*.

[36] Staud R, Robinson ME, Weyl EE, Price DD (2010). Pain variability in fibromyalgia is related to activity and rest: role of peripheral tissue impulse input. *Journal of Pain*.

[37] Staud R, Nagel S, Robinson ME, Price DD (2009). Enhanced central pain processing of fibromyalgia patients is maintained by muscle afferent input: a randomized, double-blind, placebo-controlled study. *Pain*.

[38] Ambalavanar R, Dessem D, Moutanni A (2006). Muscle inflammation induces a rapid increase in calcitonin gene-related peptide (CGRP) mRNA that temporally relates to CGRP immunoreactivity and nociceptive behavior. *Neuroscience*.

[39] Imbe H, Dubner R, Ren K (1999). Masseteric inflammation-induced Fos protein expression in the trigeminal interpolaris/caudalis transition zone: contribution of somatosensory-vagal-adrenal integration. *Brain Research*.

[40] Ro JY (2005). Bite force measurement in awake rats: a behavioral model for persistent orofacial muscle pain and hyperalgesia. *Journal of Orofacial Pain*.

[41] Sprott H, Salemi S, Gay RE (2004). Increased DNA fragmentation and ultrastructural changes in fibromyalgic muscle fibres. *Annals of the Rheumatic Diseases*.

[42] Stohler CS, Kowalski CJ, Lund JP (2001). Muscle pain inhibits cutaneous touch perception. *Pain*.

[43] Svensson P, Arendt-Nielsen L, Houe L (1998). Muscle pain modulates mastication: an experimental study in humans. *Journal of Orofacial Pain*.

[44] Ro JY, Capra NF (2001). Modulation of jaw muscle spindle afferent activity following intramuscular injections with hypertonic saline. *Pain*.

[45] Tegeder I, Zimmermann J, Meller ST, Geisslinger G (2002). Release of algesic substances in human experimental muscle pain. *Inflammation Research*.

[46] Iggo A (1960). Non-myelinated afferent fibers from mammalian skeletal muscle. *Journal of Physiology*.

[47] Hoheisel U, Reinöhl J, Unger T, Mense S (2004). Acidic pH and capsaicin activate mechanosensitive group IV muscle receptors in the rat. *Pain*.

[48] Sluka KA, Kalra A, Moore SA (2001). Unilateral intramuscular injections of acidic saline produce a bilateral, long-lasting hyperalgesia. *Muscle and Nerve*.

[49] Sluka KA, Rohlwing JJ, Bussey RA, Eikenberry SA, Wilken JM (2002). Chronic muscle pain induced by repeated acid injection is reversed by spinally administered mu- and delta-, but not kappa-, opioid receptor agonists. *Journal of Pharmacology and Experimental Therapeutics*.

[50] Skyba DA, King EW, Sluka KA (2002). Effects of NMDA and non-NMDA ionotropic glutamate receptor antagonists on the development and maintenance of hyperalgesia induced by repeated intramuscular injection of acidic saline. *Pain*.

[51] Ambalavanar R, Yallampalli C, Yallampalli U, Dessem D (2007). Injection of adjuvant but not acidic saline into craniofacial muscle evokes nociceptive behaviors and neuropeptide expression. *Neuroscience*.

[52] Connor M, Naves LA, McCleskey EW (2005). Contrasting phenotypes of putative proprioceptive and nociceptive trigeminal neurons innervating jaw muscle in rat. *Molecular Pain*.

[53] Diehl B, Hoheisel U, Mense S (1993). The influence of mechanical stimuli and of acetylsalicylic acid on the discharges of slowly conducting afferent units from normal and inflamed muscle in the rat. *Experimental Brain Research*.

[54] Kehl LJ, Trempe TM, Hargreaves KM (2000). A new animal model for assessing mechanisms and management of muscle hyperalgesia. *Pain*.

[55] Radhakrishnan R, Moore SA, Sluka KA (2003). Unilateral carrageenan injection into muscle or joint induces chronic bilateral hyperalgesia in rats. *Pain*.

[56] Kaneyama K, Segami N, Nishimura M, Suzuki T, Sato J (2002). Importance of proinflammatory cytokines in synovial fluid from 121 joints with temporomandibular disorders. *British Journal of Oral and Maxillofacial Surgery*.

[57] Kaneyama K, Segami N, Sun W, Sato J, Fujimura K (2005). Analysis of tumor necrosis factor-*α*, interleukin-6, interleukin-1*β*, soluble tumor necrosis factor receptors I and II, interleukin-6 soluble receptor, interleukin-1 soluble receptor type II, interleukin-1 receptor antagonist, and protein in the synovial fluid of patients with temporomandibular joint disorders. *Oral Surgery, Oral Medicine, Oral Pathology, Oral Radiology and Endodontology*.

[58] Salemi S, Rethage J, Wollina U (2003). Detection of interleukin 1*β* (IL-1*β*), IL-6, and tumor necrosis factor-*α* in skin of patients with fibromyalgia. *Journal of Rheumatology*.

[59] Haas DA, Nakanishi O, MacMillan RE, Jordan RC, Hu JW (1992). Development of an orofacial model of acute inflammation in the rat. *Archives of Oral Biology*.

[60] Yu XM, Sessle BJ, Vernon H, Hu JW (1995). Effects of inflammatory irritant application to the rat temporomandibular joint on jaw and neck muscle activity. *Pain*.

[61] Hartwig AC, Mathias SI, Law AS, Gebhart GF (2003). Characterization and opioid modulation of inflammatory temporomandibular joint pain in the rat. *Journal of Oral and Maxillofacial Surgery*.

[62] Ro JY, Harriott A, Crouse U, Capra NF (2003). Innocuous jaw movements increase c-fos expression in trigeminal sensory nuclei produced by masseter muscle inflammation. *Pain*.

[63] Petersen M, LaMotte RH (1991). Relationships between capsaicin sensitivity of mammalian sensory neurons, cell size and type of voltage gated Ca-currents. *Brain Research*.

[64] Takeda M, Tanimoto T, Ito M, Nasu M, Matsumoto S (2005). Role of capsaicin-sensitive primary afferent inputs from the masseter muscle in the C spinal neurons responding to tooth-pulp stimulation in rats. *Experimental Brain Research*.

[65] Bautista DM, Jordt SE, Nikai T (2006). TRPA1 mediates the inflammatory actions of environmental irritants and proalgesic agents. *Cell*.

[66] Kobayashi K, Fukuoka T, Obata K (2005). Distinct expression of TRPM8, TRPA1, and TRPV1 mRNAs in rat primary afferent neurons with A*δ*/C-fibers and colocalization with Trk receptors. *Journal of Comparative Neurology*.

[67] Simone DA, Marchettini P, Caputi G, Ochoa JL (1994). Identification of muscle afferents subserving sensation of deep pain in humans. *Journal of Neurophysiology*.

[68] Marchettini P, Simone DA, Caputi G, Ochoa JL (1996). Pain from excitation of identified muscle nociceptors in humans. *Brain Research*.

[69] Cairns BE, Gambarota G, Svensson P, Arendt-Nielsen L, Berde CB (2002). Glutamate-induced sensitization of rat masseter muscle fibers. *Neuroscience*.

[70] Ernberg M, Lundeberg T, Kopp S (2000). Pain and allodynia/hyperalgesia induced by intramuscular injection of serotonin in patients with fibromyalgia and healthy individuals. *Pain*.

[71] Fock S, Mense S (1976). Excitatory effects of 5 hydroxytryptamine, histamine and potassium ions on muscular group IV afferent units: a comparison with bradykinin. *Brain Research*.

[72] Hoheisel U, Unger T, Mense S (2005). Excitatory and modulatory effects of inflammatory cytokines and neurotrophins on mechanosensitive group IV muscle afferents in the rat. *Pain*.

[73] Mørk H, Ashina M, Bendtsen L, Olesen J, Jensen R (2003). Experimental muscle pain and tenderness following infusion of endogenous substances in humans. *European Journal of Pain*.

[74] Franz M, Mense S (1975). Muscle receptors with group IV afferent fibres responding to application of bradykinin. *Brain Research*.

[75] Mense S (1981). Sensitization of group IV muscle receptors to bradykinin by 5-hydroxytryptamine and prostaglandin E_2_. *Brain Research*.

[76] Schneider S, Randoll D, Buchner M (2006). Why do women have back pain more than men? A representative prevalence study in the Federal Republic of Germany. *Clinical Journal of Pain*.

[77] Svensson P, Cairns BE, Wang K, Arendt-Nielsen L (2003). Injection of nerve growth factor into human masseter muscle evokes long-lasting mechanical allodynia and hyperalgesia. *Pain*.

[78] Kessler W, Kirchhoff C, Reeh PW, Handwerker HO (1992). Excitation of cutaneous afferent nerve endings in vitro by a combination of inflammatory mediators and conditioning effect of substance P. *Experimental Brain Research*.

[79] Lovering RM, De Deyne PG (2004). Contractile function, sarcolemma integrity, and the loss of dystrophin after skeletal muscle eccentric contraction-induced injury. *American Journal of Physiology*.

[80] Lovering RM, Hakim M, Moorman CT, De Deyne PG (2005). The contribution of contractile pre-activation to loss of function after a single lengthening contraction. *Journal of Biomechanics*.

[81] Dessem D, Ambalavanar R, Evancho M, Moutanni A, Yallampalli C, Bai G (2010). Eccentric muscle contraction and stretching evoke mechanical hyperalgesia and modulate CGRP and P2X_3_ expression in a functionally relevant manner. *Pain*.

[82] Beaton LJ, Tarnopolsky MA, Phillips SM (2002). Contraction-induced muscle damage in humans following calcium channel blocker administration. *Journal of Physiology*.

[83] Nosaka K, Newton M, Sacco P (2002). Delayed-onset muscle soreness does not reflect the magnitude of eccentric exercise-induced muscle damage. *Scandinavian Journal of Medicine and Science in Sports*.

[84] Pizza FX, Peterson JM, Baas JH, Koh TJ (2005). Neutrophils contribute to muscle injury and impair its resolution after lengthening contractions in mice. *Journal of Physiology*.

[85] Taguchi T, Matsuda T, Tamura R, Sato J, Mizumura K (2005). Muscular mechanical hyperalgesia revealed by behavioural pain test and c-Fos expression in the spinal dorsal horn after eccentric contraction in rats. *Journal of Physiology*.

[86] McLoughlin TJ, Mylona E, Hornberger TA, Esser KA, Pizza FX (2003). Inflammatory cells in rat skeletal muscle are elevated after electrically stimulated contractions. *Journal of Applied Physiology*.

[87] Lieber RL, Thornell LE, Fridén J (1996). Muscle cytoskeletal disruption occurs within the first 15 min of cyclic eccentric contraction. *Journal of Applied Physiology*.

[88] Lapointe BM, Frémont P, Côté CH (2002). Adaptation to lengthening contractions is independent of voluntary muscle recruitment but relies on inflammation. *American Journal of Physiology*.

[89] Chen YW, Hubal MJ, Hoffman EP, Thompson PD, Clarkson PM (2003). Molecular responses of human muscle to eccentric exercise. *Journal of Applied Physiology*.

[90] Tsivitse SK, McLoughlin TJ, Peterson JM, Mylona E, McGregor SJ, Pizza FX (2003). Downhill running in rats: influence on neutrophils, macrophages, and MyoD+ cells in skeletal muscle. *European Journal of Applied Physiology*.

[91] Proske U, Allen TJ (2005). Damage to skeletal muscle from eccentric exercise. *Exercise and Sport Sciences Reviews*.

[92] Warren GL, Summan M, Gao X, Chapman R, Hulderman T, Simeonova PP (2007). Mechanisms of skeletal muscle injury and repair revealed by gene expression studies in mouse models. *Journal of Physiology*.

[93] Jensen L, Pilegaard H, Neufer PD, Hellsten Y (2004). Effect of acute exercise and exercise training on VEGF splice variants in human skeletal muscle. *American Journal of Physiology*.

[94] Jonhagen S, Ackermann P, Saartok T, Renstrom PA (2006). Calcitonin gene related peptide and neuropeptide Y in skeletal muscle after eccentric exercise: a microdialysis study. *British Journal of Sports Medicine*.

[95] Kivelä R, Silvennoinen M, Lehti M, Jalava S, Vihko V, Kainulainen H (2008). Exercise-induced expression of angiogenic growth factors in skeletal muscle and in capillaries of healthy and diabetic mice. *Cardiovascular Diabetology*.

[96] Armstrong RB, Ogilvie RW, Schwane JA (1983). Eccentric exercise-induced injury to rat skeletal muscle. *Journal of Applied Physiology Respiratory Environmental and Exercise Physiology*.

[97] Darr KC, Schultz E (1987). Exercise-induced satellite cell activation in growing and mature skeletal muscle. *Journal of Applied Physiology*.

[98] Pavlath GK, Thaloor D, Rando TA, Cheong M, English AW, Zheng B (1998). Heterogeneity among muscle precursor cells in adult skeletal muscles with differing regenerative capacities. *Developmental Dynamics*.

[99] Shortreed K, Johnston A, Hawke T, Tiidus P (2007). Satellite cells and muscle repair. *Skeletal Muscle Damage and Repair*.

[100] Hill M, Wernig A, Goldspink G (2003). Muscle satellite (stem) cell activation during local tissue injury and repair. *Journal of Anatomy*.

[101] Saka T, Akova B, Yazici Z, Sekir U, Gur H, Ozarda Y (2009). Diference in the magnitude of muscle damage between elbow flexors and knee extensors eccentric exercises. *Journal of Sports Science and Medicine*.

[102] Jamurtas AZ, Theocharis V, Tofas T (2005). Comparison between leg and arm eccentric exercises of the same relative intensity on indices of muscle damage. *European Journal of Applied Physiology*.

[103] Juhlin L, Evers H, Broberg F (1980). A lidocaine-prilocaine cream for superficial skin surgery and painful lesions. *Acta Dermato-Venereologica*.

[104] Ambalavanar R, Moritani M, Moutanni A, Gangula P, Yallampalli C, Dessem D (2006). Deep tissue inflammation upregulates neuropeptides and evokes nociceptive behaviors which are modulated by a neuropeptide antagonist. *Pain*.

[105] Lovering RM, Roche JA, Bloch RJ, De Deyne PG (2007). Recovery of function in skeletal muscle following 2 different contraction-induced injuries. *Archives of Physical Medicine and Rehabilitation*.

[106] Dessem D, Moritani M, Ambalavanar R (2007). Nociceptive craniofacial muscle primary afferent neurons synapse in both the rostral and caudal brain stem. *Journal of Neurophysiology*.

[107] Evancho M, Ambalavanar R, Dessem D Masseter muscle exhibits impaired repair following eccentric contraction of the muscle.

[108] McBride TA, Gorin FA, Carlsen RC (1995). Prolonged recovery and reduced adaptation in aged rat muscle following eccentric exercise. *Mechanisms of Ageing and Development*.

[109] Brooks SV, Zerba E, Faulkner JA (1995). Injury to muscle fibres after single stretches of passive and maximally stimulated muscles in mice. *Journal of Physiology*.

[110] Faulkner JA, Brooks SV, Opiteck JA (1993). Injury to skeletal muscle fibers during contractions: conditions of occurrence and prevention. *Physical Therapy*.

[111] Warren GL, Lowe DA, Armstrong RB (1999). Measurement tools used in the study of eccentric contraction-induced injury. *Sports Medicine*.

[112] Newham DJ, Jones DA, Clarkson PM (1987). Repeated high-force eccentric exercise: effects on muscle pain and damage. *Journal of Applied Physiology*.

[113] Sacco P, Jones DA (1992). The protective effect of damaging eccentric exercise against repeated bouts of exercise in the mouse tibialis anterior muscle. *Experimental physiology*.

[114] Pizza FX, Davis BH, Henrickson SD (1996). Adaptation to eccentric exercise: effect on CD64 and CD 11b/CD 18 expression. *Journal of Applied Physiology*.

[115] McKune AJ, Smith LL, Semple SJ, Mokethwa B, Wadee AA (2006). Immunoglobulin responses to a repeated bout of downhill running. *British Journal of Sports Medicine*.

[116] Chen TC, Chen HL, Lin MJ, Wu CJ, Nosaka K (2009). Muscle damage responses of the elbow flexors to four maximal eccentric exercise bouts performed every 4 weeks. *European Journal of Applied Physiology*.

[117] Paulsen G, Lauritzen F, Bayer ML (2009). Subcellular movement and expression of HSP27, alphaB-crystallin, and HSP70 after two bouts of eccentric exercise in humans. *Journal of Applied Physiology*.

[118] McHugh MP (2003). Recent advances in the understanding of the repeated bout effect: the protective effect against muscle damage from a single bout of eccentric exercise. *Scandinavian Journal of Medicine and Science in Sports*.

[119] Lynn R, Morgan DL (1994). Decline running produces more sarcomeres in rat vastus intermedius muscle fibers than does incline running. *Journal of Applied Physiology*.

[120] Lynn R, Talbot JA, Morgan DL (1998). Differences in rat skeletal muscles after incline and decline running. *Journal of Applied Physiology*.

[121] Brockett CL, Morgan DL, Proske U (2001). Human hamstring muscles adapt to eccentric exercise by changing optimum length. *Medicine and Science in Sports and Exercise*.

[122] Ingalls CP, Warren GL, Williams JH, Ward CW, Armstrong RB (1998). E-C coupling failure in mouse EDL muscle after in vivo eccentric contractions. *Journal of Applied Physiology*.

[123] Pizza FX, Koh TJ, McGregor SJ, Brooks SV (2002). Muscle inflammatory cells after passive stretches, isometric contractions, and lengthening contractions. *Journal of Applied Physiology*.

[124] Hubal MJ, Chen TC, Thompson PD, Clarkson PM (2008). Inflammatory gene changes associated with the repeated-bout effect. *American Journal of Physiology*.

[125] Lehti TM, Kalliokoski R, Komulainen J (2007). Repeated bout effect on the cytoskeletal proteins titin, desmin, and dystrophin in rat skeletal muscle. *Journal of Muscle Research and Cell Motility*.

[126] Koh TJ, Brooks SV (2001). Lengthening contractions are not required to induce protection from contraction-induced muscle injury. *American Journal of Physiology*.

[127] Reich TE, Lindstedt SL, LaStayo PC, Pierotti DJ (2000). Is the spring quality of muscle plastic?. *American Journal of Physiology*.

[128] McHugh MP, Connolly DAJ, Eston RG, Kremenic IJ, Nicholas SJ, Gleim GW (1999). The role of passive muscle stiffness in symptoms of exercise-induced muscle damage. *American Journal of Sports Medicine*.

[129] Marqueste T, Giannesini B, Fur YL, Cozzone PJ, Bendahan D (2008). Comparative MRI analysis of T2 changes associated with single and repeated bouts of downhill running leading to eccentric-induced muscle damage. *Journal of Applied Physiology*.

[130] Glaros AG, Burton E (2004). Parafunctional clenching, pain, and effort in temporomandibular disorders. *Journal of Behavioral Medicine*.

[131] Glaros AG, Williams K, Lausten L (2005). The role of parafunctions, emotions and stress in predicting facial pain. *Journal of the American Dental Association*.

[132] Arima T, Svensson P, Arendt-Nielsen L (1999). Experimental grinding in healthy subjects: a model for postexercise jaw muscle soreness?. *Journal of Orofacial Pain*.

[133] Christensen LV (1971). Facial pain and internal pressure of masseter muscle in experimental bruxism in man. *Archives of Oral Biology*.

[134] Bowley JF, Gale EN (1987). Experimental masticatory muscle pain. *Journal of Dental Research*.

[135] Clark GT, Jow RW, Lee JJ (1989). Jaw pain and stiffness levels after repeated maximum voluntary clenching. *Journal of dental research*.

[136] Svensson P, Houe L, Arendt-Nielsen L (1997). Effect of systemic versus topical nonsteroidal anti-inflammatory drugs on postexercise jaw-muscle soreness: a placebo-controlled study. *Journal of Orofacial Pain*.

[137] Newham DJ, McPhail G, Mills KR, Edwards RH (1983). Ultrastructural changes after concentric and eccentric contractions of human muscle. *Journal of the Neurological Sciences*.

[138] Wiesmann C, Muller YA, De Vos AM (2000). Ligand-binding sites in Ig-like domains of receptor tyrosine kinases. *Journal of Molecular Medicine*.

[139] Xie Y, Tisi MA, Yeo TT, Longo FM (2000). Nerve growth factor (NGF) loop 4 dimeric mimetics activate ERK and AKT and promote NGF-like neurotrophic effects. *Journal of Biological Chemistry*.

[140] Dyck PJ, Peroutka S, Rask C (1997). Intradermal recombinant human nerve growth factor induces pressure allodynia and lowered heat-pain threshold in humans. *Neurology*.

[141] Wild KD, Bian D, Zhu D (2007). Antibodies to nerve growth factor reverse established tactile allodynia in rodent models of neuropathic pain without tolerance. *Journal of Pharmacology and Experimental Therapeutics*.

[142] Woolf CJ, Safieh-Garabedian B, Ma QP, Crilly P, Winter J (1994). Nerve growth factor contributes to the generation of inflammatory sensory hypersensitivity. *Neuroscience*.

[143] Woolf CJ, Ma QP, Allchorne A, Poole S (1996). Peripheral cell types contributing to the hyperalgesic action of nerve growth factor in inflammation. *Journal of Neuroscience*.

[144] Kehl L, Velly A, Besspiata D, Jackson A, Lenton P, Schiffman E (2008). Characteristics of muscle pain are associated with nerve growth factor content in human muscle. *Society for Neuroscience Abstracts*.

[145] Dessem D, Mouotani A, Cleiman R (2010). Sex differences in movement-induced muscle pain. *Society for Neuroscience Abstracts*.

[146] Murase S, Terazawa E, Queme F (2010). Bradykinin and nerve growth factor play pivotal roles in muscular mechanical hyperalgesia after exercise (d
elayed-onset muscle soreness). *Journal of Neuroscience*.

[147] Wu C, Erickson MA, Xu J, Wild KD, Brennan TJ (2009). Expression profile of nerve growth factor after muscle incision in the rat. *Anesthesiology*.

[148] Petty BG, Cornblath DR, Adornato BT (1994). The effect of systemically administered recombinant human nerve growth factor in healthy human subjects. *Annals of Neurology*.

[149] Taguchi T, Sato J, Mizumura K (2005). Augmented mechanical response of muscle thin-fiber sensory receptors recorded from rat muscle-nerve preparations in vitro after eccentric contraction. *Journal of Neurophysiology*.

[150] Toti P, Villanova M, Vatti R (2003). Nerve growth factor expression in human dystrophic muscles. *Muscle and Nerve*.

[151] Murphy RA, Singer RH, Saide JD (1977). Synthesis and secretion of a high molecular weight form of nerve growth factor by skeletal muscle cells in culture. *Proceedings of the National Academy of Sciences of the United States of America*.

[152] Di Marco E, Marchisio PC, Bondanza S, Franzi AT, Cancedda R, De Luca M (1991). Growth-regulated synthesis and secretion of biologically active nerve growth factor by human keratinocytes. *Journal of Biological Chemistry*.

[153] Olgart C, Frossard N (2001). Human lung fibroblasts secrete nerve growth factor: effect of inflammatory cytokines and glucocorticoids. *European Respiratory Journal*.

[154] Leon A, Buriani A, Toso RD (1994). Mast cells synthesize, store, and release nerve growth factor. *Proceedings of the National Academy of Sciences of the United States of America*.

[155] Nilsson G, Forsberg-Nilsson K, Xiang Z, Hallböök F, Nilsson K, Metcalfe DD (1997). Human mast cells express functional TrkA and are a source of nerve growth factor. *European Journal of Immunology*.

[156] Stanzel RDP, Lourenssen S, Blennerhassett MG (2008). Inflammation causes expression of NGF in epithelial cells of the rat colon. *Experimental Neurology*.

[157] D’Arco M, Giniatullin R, Simonetti M (2007). Neutralization of nerve growth factor induces plasticity of ATP-sensitive P2X_3_ receptors of nociceptive trigeminal ganglion neurons. *Journal of Neuroscience*.

[158] Supowit SC, Zhao H, DiPette DJ (2001). Nerve growth factor enhances calcitonin gene-related peptide expression in the spontaneously hypertensive rat. *Hypertension*.

[159] Ramer MS, Bradbury EJ, McMahon SB (2001). Nerve growth factor induces P2X_3_ expression in sensory neurons. *Journal of Neurochemistry*.

[160] Xing J, Lu J, Li J (2009). Contribution of nerve growth factor to augmented TRPV1 responses of muscle sensory neurons by femoral artery occlusion. *American Journal of Physiology*.

[161] Orita S, Ohtori S, Nagata M (2010). Inhibiting nerve growth factor or its receptors downregulates calcitonin gene-related peptide expression in rat lumbar dorsal root ganglia innervating injured intervertebral discs. *Journal of Orthopaedic Research*.

[162] Dunn PM, Zhong YU, Burnstock G (2001). P2X receptors in peripheral neurons. *Progress in Neurobiology*.

[163] Khakh BS, North RA (2006). P2X receptors as cell-surface ATP sensors in health and disease. *Nature*.

[164] Reinöhl J, Hoheisel U, Unger T, Mense S (2003). Adenosine triphosphate as a stimulant for nociceptive and non-nociceptive muscle group IV receptors in the rat. *Neuroscience Letters*.

[165] Shinoda M, Ozaki N, Sugiura Y (2008). Involvement of ATP and its receptors on nociception in rat model of masseter muscle pain. *Pain*.

[166] North RA (2003). The P2X_3_ subunit: a molecular target in pain therapeutics. *Current Opinion in Investigational Drugs*.

[167] Ambalavanar R, Dessem D (2009). Emerging peripheral receptor targets for deep-tissue craniofacial pain therapies. *Journal of Dental Research*.

[168] Ambalavanar R, Moritani M, Dessem D (2005). Trigeminal P2X_3_ receptor expression differs from dorsal root ganglion and is modulated by deep tissue inflammation. *Pain*.

[169] Cook SP, McCleskey EW (2002). Cell damage excites nociceptors through release of cytosolic ATP. *Pain*.

[170] Stewart LC, Deslauriers R, Kupriyanov VV (1994). Relationships between cytosolic [ATP], [ATP]/[ADP] and ionic fluxes in the perfused rat heart: a 31P, 23Na and 87Rb NMR study. *Journal of Molecular and Cellular Cardiology*.

[171] Gamse R, Posch M, Saria A, Jancsó G (1987). Several mediators appear to interact in neurogenic inflammation. *Acta Physiologica Hungarica*.

[172] Nahin RL, Byers MR (1994). Adjuvant-induced inflammation of rat paw is associated with altered calcitonin gene-related peptide immunoreactivity within cell bodies and peripheral endings of primary afferent neurons. *Journal of Comparative Neurology*.

[173] Woolf C, Wiesenfeld-Hallin Z (1986). Substance P and calcitonin gene-related peptide synergistically modulate the gain of the nociceptive flexor withdrawal reflex in the rat. *Neuroscience Letters*.

[174] Zhang L, Hoff AO, Wimalawansa SJ, Cote GJ, Gagel RF, Westlund KN (2001). Arthritic calcitonin/*α* calcitonin gene-related peptide knockout mice have reduced nociceptive hypersensitivity. *Pain*.

[175] Hutchins B, Spears R, Hinton RJ, Harper RP (2000). Calcitonin gene-related peptide and substance P immunoreactivity in rat trigeminal ganglia and brainstem following adjuvant-induced inflammation of the temporomandibular joint. *Archives of Oral Biology*.

[176] Ohlen A, Lindbom L, Staines W (1987). Substance P and calcitonin gene-related peptide: immunohistochemical localisation and microvascular effects in rabbit skeletal muscle. *Naunyn-Schmiedeberg's Archives of Pharmacology*.

[177] Brain SD, Tippins JR, Morris HR (1986). Potent vasodilator activity of calcitonin gene-related peptide in human skin. *Journal of Investigative Dermatology*.

[178] Bulling DG, Kelly D, Bond S, McQueen DS, Seckl JR (2001). Adjuvant-induced joint inflammation causes very rapid transcription of beta-preprotachykinin and alpha-CGRP genes in innervating sensory ganglia. *Journal of Neurochemistry*.

[179] Bradbury EJ, Burnstock G, McMahon SB (1998). The expression of P2X_3_ purinoreceptors in sensory neurons: effects of axotomy and glial-derived neurotrophic factor. *Molecular and Cellular Neurosciences*.

[180] Petruska JC, Napaporn J, Johnson RD, Gu JG, Cooper BY (2000). Subclassified acutely dissociated cells of rat DRG: histochemistry and patterns of capsaicin-, proton-, and ATP-activated currents. *Journal of Neurophysiology*.

[181] Price TJ, Louria MD, Candelario-Soto D (2005). Treatment of trigeminal ganglion neurons in vitro with NGF, GDNF or BDNF: effects on neuronal survival, neurochemical properties and TRPV1-mediated neuropeptide secretion. *BMC Neuroscience*.

[182] Simonetti M, Fabbro A, D'Arco M (2006). Comparison of P2X and TRPV1 receptors in ganglia or primary culture of trigeminal neurons and their modulation by NGF or serotonin. *Molecular Pain*.

[183] Kanehira H, Agariguchi A, Kato H, Yoshimine S, Inoue H (2008). Association between stress and temporomandibular disorder. *Nihon Hotetsu Shika Gakkai Zasshi*.

[184] Licini F, Nojelli A, Segù M, Collesano V (2009). Role of psychosocial factors in the etiology of temporomandibular disorders: relevance of a biaxial diagnosis. *Minerva Stomatologica*.

[185] Nilsson AM, Dahlstrm L (2010). Perceived symptoms of psychological distress and salivary cortisol levels in young women with muscular or disk-related temporomandibular disorders. *Acta Odontologica Scandinavica*.

[186] Martínez-Lavín M, Hermosillo AG, Rosas M, Soto ME (1998). Circadian studies of autonomic nervous balance in patients with fibromyalgia: a heart rate variability analysis. *Arthritis and Rheumatism*.

[187] Schmidt JE, Carlson CR (2009). A controlled comparison of emotional reactivity and physiological response in masticatory muscle pain patients. *Journal of Orofacial Pain*.

[188] Mizurma K, Taguchi T, Nasu T (2007). Persistent muscular mechanical hyperalgesia induced by lengthening contraction followed by repetitive stress. *Journal of Musculoskeletal Pain*.

[189] Staud R, Cannon RC, Mauderli AP, Robinson ME, Price DD, Vierck CJ (2003). Temporal summation of pain from mechanical stimulation of muscle tissue in normal controls and subjects with fibromyalgia syndrome. *Pain*.

[190] Jarvis MF, Burgard EC, McGaraughty S (2002). A-317491, a novel potent and selective non-nucleotide antagonist of P2X_3_ and P2X_3_ receptors, reduces chronic inflammatory and neuropathic pain in the rat. *Proceedings of the National Academy of Sciences of the United States of America*.

[191] Hefti FF, Rosenthal A, Walicke PA (2006). Novel class of pain drugs based on antagonism of NGF. *Trends in Pharmacological Sciences*.

[192] Watson JJ, Allen SJ, Dawbarn D (2008). Targeting nerve growth factor in pain: what is the therapeutic potential?. *BioDrugs*.

